# Recurrent laryngeal nerve’s course running anteriorly to a thyroid tumor

**DOI:** 10.1186/s13044-023-00172-6

**Published:** 2023-07-20

**Authors:** Minoru Kihara, Akira Miyauchi, Makoto Fujishima, Tomo Ishizaka, Akihide Matsunaga, Shiori Kawano, Masashi Yamamoto, Takahiro Sasaki, Hiroo Masuoka, Takuya Higashiyama, Yasuhiro Ito, Naoyoshi Onoda, Akihiro Miya, Takashi Akamizu

**Affiliations:** 1 Department of Surgery Kuma Hospital, 8-2-35 Shimoyamate-Dori, Chuo-Ku, Kobe, Hyogo 650-0011 Japan; 2 Department of Head and Neck Surgery Kuma Hospital, 8-2-35 Shimoyamate-Dori, Chuo-Ku, Kobe, Hyogo 650-0011 Japan; 3 Department of Internal Medicine, Kuma Hospital, 8-2-35 Shimoyamate-Dori, Chuo-Ku, Kobe, Hyogo 650-0011 Japan

**Keywords:** Recurrent laryngeal nerve, RLN running course, Thyroidectomy, Intraoperative neuromonitoring

## Abstract

The thyroid gland's neurovascular relationship is commonly portrayed as the recurrent laryngeal nerve (RLN) coursing posteriorly to the thyroid gland. We report a rare case with the RLN running anteriorly to a thyroid tumor. A 56-year-old Japanese woman underwent a thyroidectomy for a benign thyroid tumor. Preoperatively, computed tomography confirmed that part of the tumor had extended into the mediastinum and was descending posteriorly up to the brachiocephalic artery. Intraoperatively, when the sternothyroid muscle was incised to expose the thyroid gland, a cord (nerve)-like structure was observed directly anterior to the thyroid tumor. Although the course of this cord-like structure was clearly different from the "traditional" course of the right RLN, the possibility that the structure was the RLN could not be excluded. The structure was traced back in order to preserve it; we saw that it entered the larynx at the lower margin of the cricothyroid muscle and approximately at the level of the cricothyroid junction through the back of the normal thyroid tissue. With intraoperative neuromonitoring, the structure was identified as the RLN. As a result, the course of the RLN run anterior to the tumor but then posterior to the 'normal thyroid' i.e. into it normal anatomical position. Had we assumed that the RLN was behind the thyroid tumor, we would have damaged the RLN. It would not be possible to diagnose this abnormal running course of the RLN reliably before surgery, but extra care should be taken in similar cases, that is, when a large thyroid tumor is descending posteriorly up to the brachiocephalic artery on the right side.

## Introduction

Recurrent laryngeal nerve (RLN) palsy is one of the most serious potential complications after thyroid surgery. The reported incidences of temporary and permanent palsy after thyroid surgery are 3.4%–7.2% and 0.2%–1.0%, respectively, based on the number of nerves at risk [1–3]. One of the reasons for this risk is variations of the RLN's anatomy. Anatomical anomalies can make it difficult to locate the RLN during thyroid surgery [4–7], and thyroid surgeons thus need complete and accurate knowledge of thyroid gland embryology, anatomy, and the relationships between the thyroid and other tissues in order to perform thyroidectomies safely. The use of intraoperative neuromonitoring (IONM) has reduced the incidence of injury to the RLN [8, 9].

The RLN's neurovascular relationship is commonly portrayed in textbooks and the literature as the RLN coursing posteriorly to the thyroid gland [10–12]. We encountered a patient whose RLN was anterior to the thyroid tumor. This report emphasizes the possible presence of an abnormal running course of the RLN and will help surgeons avoid accidentally cutting an RLN in thyroid surgery.

## Case presentation

**Fig. 1:**
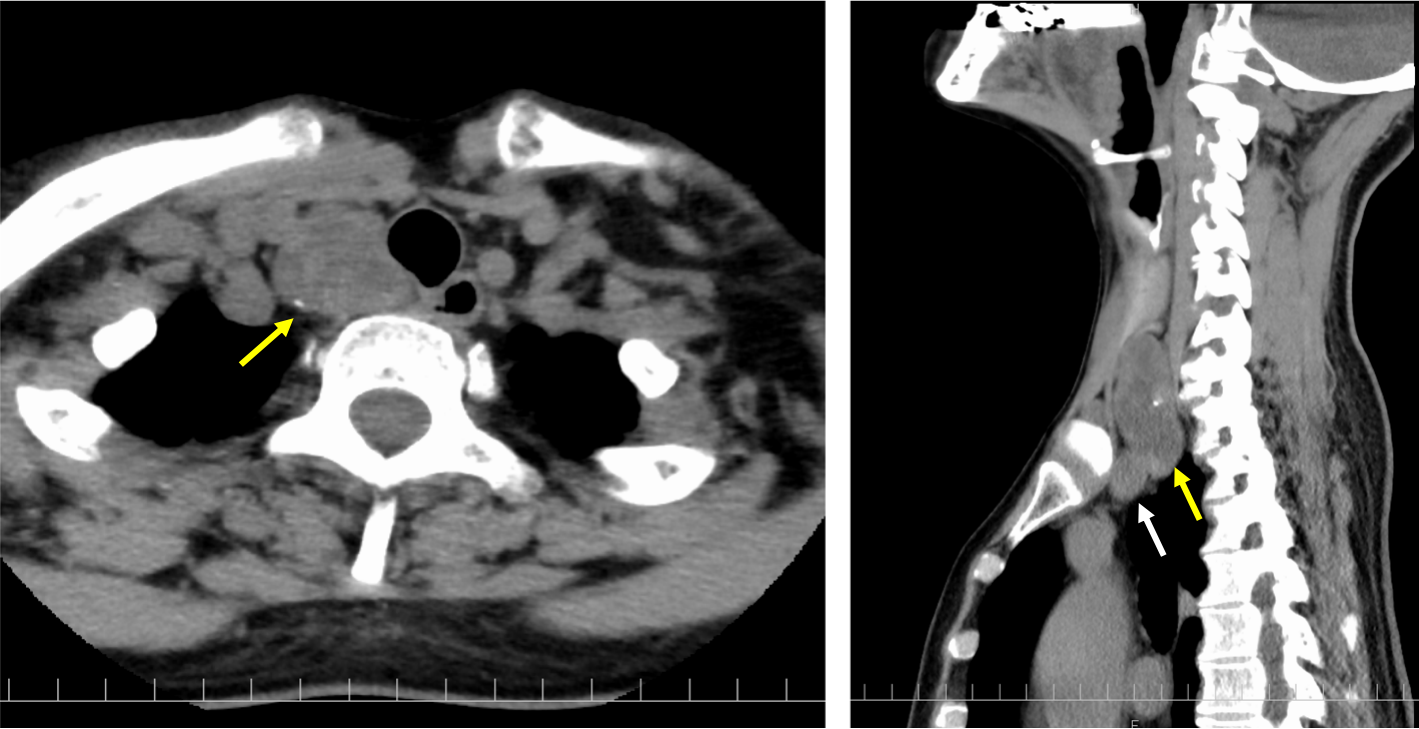
Computed tomography image at cervical extension. The tumor (*yellow arrow*) had extended into the mediastinum and was descending posteriorly into the brachiocephalic artery (*white arrow*)

A 56-year-old Japanese woman visited our hospital after a thyroid tumor was identified at another hospital. Laboratory findings showed that her thyroid status was euthyroidism; the serum level of thyroglobulin (Tg) was 102 ng/mL, and serum Tg antibody was negative. Ultrasonography revealed a tumor measuring 49 × 21 × 23 mm in the right lobe of the thyroid gland. Computed tomography (CT) at cervical extension confirmed that part of the tumor had extended into the mediastinum and was descending posteriorly up to the brachiocephalic artery (Fig. 1). Fine-needle aspiration cytology was performed under ultrasound guidance, and the tumor was identified as a benign follicular nodule (category II per the Bethesda System for reporting thyroid cytopathology [13]).

**Fig. 2:**
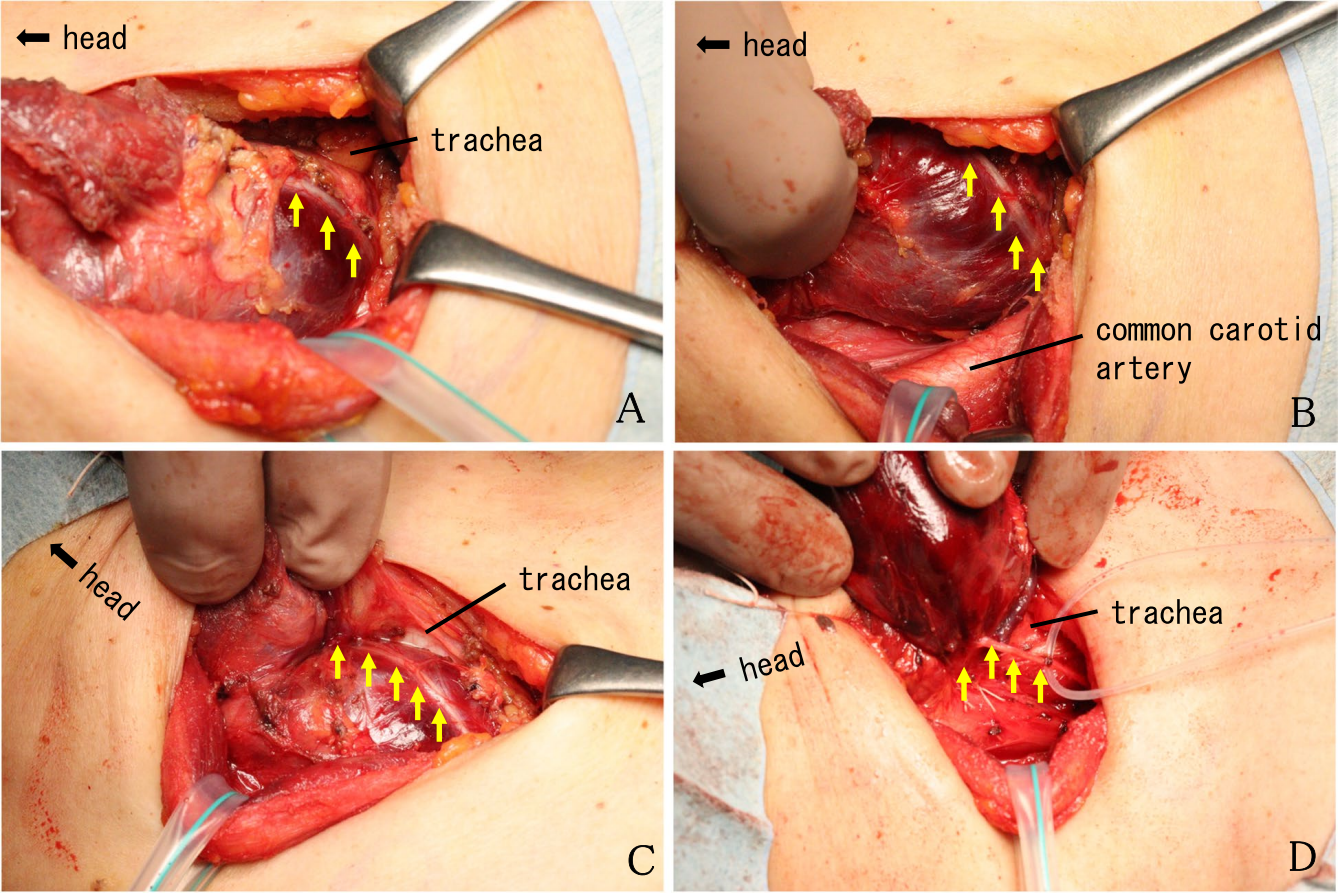
Intraoperative photos. **A**, **B** Just after the sternothyroid muscle was incised to expose the thyroid gland, the recurrent laryngeal nerve (RLN) (*arro*w) was seen anterior to the thyroid tumor. **C** The RLN ran in a course that was anterior to the thyroid tumor, toward the periphery. **D** The RLN was identified entering the trachea at the level of the cricothyroid joint

We performed a right thyroid lobectomy because of the patient's chief complaint of neck compression. Intraoperatively, after a skin-crease incision was made and the sternohyoid muscles were separated at the midline, when the sternothyroid muscle was incised to expose the thyroid gland, we observed a cord (nerve)-like structure directly anterior to the thyroid tumor (Fig. 2A,B). Although the course of this cord-like structure was clearly different from the "traditional" course of the right RLN, the possibility that the structure was the RLN could not be excluded.

IONM was performed using the nerve stimulator of nerve integrity monitoring equipment (NIM™ 3.0 IONM system, Medtronic, Jacksonville, FL, USA). We intubated the patient using a regular endotracheal tube instead of an endotracheal tube integrated with surface electrodes (NIM Flex EMG endotracheal tube, Medtronic). Because the electromyography (EMG) signal could not be obtained, it was not immediately possible to determine whether or not the cord-like structure was the RLN. We dissected the surrounding thyroid gland while preserving the structure, and we began to trace its course (Fig. 2C). Careful tissue dissection was performed in the plane between the carotid sheath and the trachea on the right side in order to identify the RLN, but the RLN in its usual anatomical position was not observed.

**Fig. 3:**
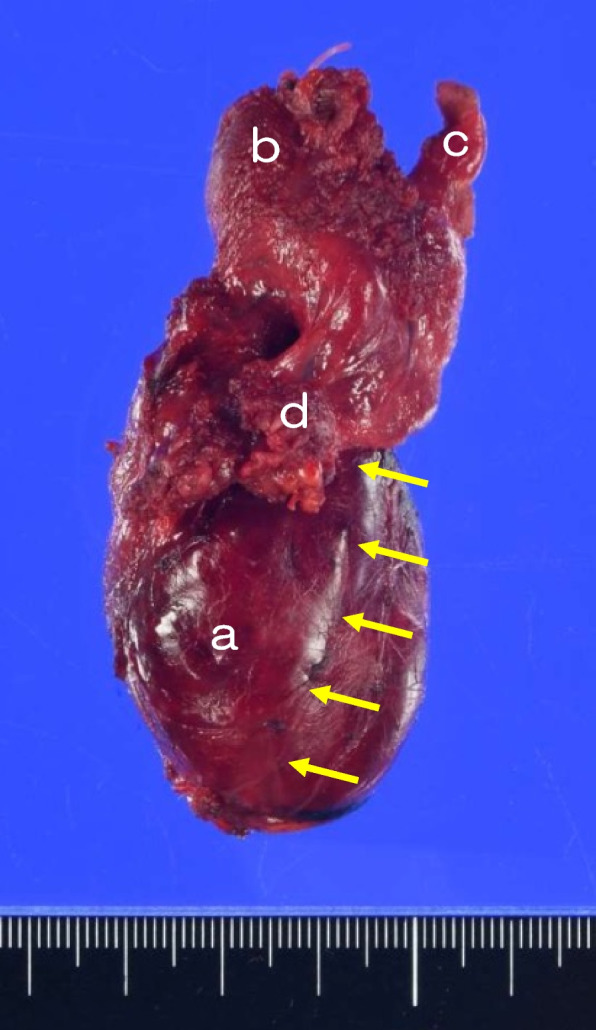
Macroscopic findings of the resected right thyroid lobe. **a** Thyroid tumor. **b** Normal thyroid tissue. **c** Pyramidal lobe. **d** The sternothyroid muscle. The right RLN ran along the *arrows*

After the right lobe of the thyroid gland was further dissected and dislocated, the preserved structure was further traced back, and it was observed to enter the larynx at the level of cricothyroid joint through the back of the normal thyroid tissue (Fig. 2D). At this stage, a finger could finally be inserted deep to the posterior lamina and fascia overlying the vertebral column. RLN neural stimulation was then performed with a nerve stimulator with the current set at 1.0 mA. After the thyroid cartilage was identified, palpation for contraction of the posterior cricoarytenoid muscle (PCA) was performed through the posterior hypopharyngeal wall. Consequently, the cord-like structure was identified as the right RLN. As a result, the course of the RLN ran anteriorly to the tumor but then posteriorly to the 'normal thyroid;' i.e., in its normal anatomical position. The surgery was completed, and both immediately after the extubation and at 2 days after surgery, laryngofiberscopy was used to visualize the patient's vocal fold mobility, and no vocal cord paralysis was observed. Macroscopic findings of the resected thyroid gland are presented in Fig. 3. The final histopathology revealed an adenomatous goiter.

## Discussion

RLN palsy can greatly diminish patients' quality of life. In addition to the hoarseness that occurs with unilateral RLN palsy, bilateral RLN palsy leads to dyspnea and often to life-threatening glottal obstruction. The preservation of the RLN is thus of great importance, and during every thyroidectomy, the intraoperative identification of the RLN is a mandatory security measure [1, 9, 14, 15]. Although the relationship between the RLN and the inferior thyroid artery is highly variable, the RLN usually runs posteriorly to the thyroid gland [4, 6, 7, 11, 12]. If the RLN is not found in the usual location, surgeons should consider the possibility of a non-RLN structure as an anatomic variation of the RLN [4, 11, 12]. The RLN has also been reported to run posteriorly to the thyroid gland [11, 12].

In our patient's case, the RLN was anterior to the thyroid tumor just behind the sternothyroid muscle; this RLN running course is very rare [16]. Hisham et al. reported that an anterior course of the RLN lying on the thyroid gland can often be encountered in reoperative procedures [16]. They noted that such an anterior course could be due to a previous mobilization or growth of remnants of the gland into a position beneath the nerve after the first procedure [16]. However, our patient's case had no previous cervical surgery including the thyroid. Normally, the left RLN runs along the tracheoesophageal groove, but the right RLN branches from the right vagus in front of the right subclavian artery and turns under the artery. It ascends obliquely from the right lateral side medially to enter the larynx at Berry's ligament. Thus, a tumor arising at the dorsal portion of the right thyroid lobe that progresses caudally may descend behind the right RLN. We suspect that this is the reason why our patient's RLN ran in front of the thyroid tumor. Thyroid surgeons should be aware that depending on the location of the tumor, the RLN may be compressed and take an unexpected route.

IONM is useful in thyroidectomies [9]. Although a visual identification of the RLN remains a gold standard in thyroid surgery, the use of neuromonitoring may help not only in the identification of nerves but also in the functional preservation of nerves, and its use reduces the incidence of RLN injury [1, 5, 8, 9, 15]. In the present patient, since an endotracheal tube integrated with surface electrodes was not performed because it is relatively expensive, palpation was performed to detect contraction of the PCA (the laryngeal twitch method). This method is a simple, readily available technique for any thyroid surgeon and can be performed with a variety of handheld, disposable, and widely available nerve stimulators [1]. However, this method requires the insertion of a finger deep to the posterior lamina and fascia overlying the vertebral column.

In our patient's surgery, a finger could not be inserted until a later timepoint, and the RLN could not be reliably identified earlier. If her RLN had been assumed to be located posterior to the thyroid tumor as is the usual anatomy (without the identification of the cord-like structure as the RLN), the RLN could have been injured during the surgery.

This case report emphasizes the importance of the intraoperative confirmation of the RLN during thyroid surgery. Although it would not have been possible to diagnose this RLN's running course variation reliably before surgery, surgeons should take extra care in similar cases, i.e., when a thyroid tumor is descending posteriorly up to the brachiocephalic artery.

## Conclusion

It is dangerous to assume that the RLN is always located at the dorsal side of a thyroid tumor during surgery. Extensive care must be taken to avoid damage to the RLN during surgery. We report the present case to remind thyroid surgeons of this variation in the RLN's course so that they will be vigilant while performing thyroidectomies.

## Data Availability

The datasets used and/or analyzed during the present study are available from the corresponding author upon reasonable request.

## Abbreviations

IONM: Intraoperative neuromonitoring
RLN: Recurrent laryngeal nerve
PCA: Posterior cricoarytenoid muscle
